# National Medical Convention

**Published:** 1846-04

**Authors:** 


### NATIONAL MEDICAL CONVENTION.

We exceedingly regret to see that there is not likely to be a full representation of the medical schools of the country at the proposed convention in May. The schools of Philadelphia, it is announced, have declined sending delegates. Professor Caldwell was appointed to represent the Medical Institute of Louisville, but we are sorry to learn from him that he will not be able to attend. It is greatly to be wished that there could be a general attendance from every quarter of the Union. Good could not fail to grow out of such a convention, and we still hope that the assemblage will be large.

